# Supplementary material for: Content-rich biological network constructed by mining PubMed abstracts
Source: BMC Bioinformatics. 2004 Oct 8;5:147. doi: 10.1186/1471-2105-5-147 (PMC528731; doi:10.1186/1471-2105-5-147)
Supplement: Additional File 2 — The original results of the above study (non-essential files are deleted to keep the file size under the limit set by BMC bioinformatics). [file 1471-2105-5-147-S2.bz2 › chilibotAdditionalFile2/dip30/258ID2569363E1022/html/CDC13_CDC2.html]

 


 **CDC13** and **CDC2** 
  
Found 69 abstracts in PubMed, retrieved 30.  
 

 What does Google say? 
 PDF only 
| .edu only 

---

**Interactive relationship** (e.g. stimulation, inhibition, etc)

**Neutral relationship**- The G1 S transition is controlled by interactions of  **Cdc13**   **Cdc2**  and its stoichiometric inhibitor, Rum1.  Ref: 9652094 Biophys Chem, 1998
- Antibodies to B type cyclins  **Cdc13**  specifically immunoprecipitated  **Cdc2**   **Cdc13**  complexes with associated kinase activity from P. carinii extracts.  Ref: 10837370 Am J Respir Cell Mol Biol, 2000
- We conclude that Srw1 controls differentiation and cell cycling at least by negatively regulating Cig2 and  **Cdc13**  associated p34  **cdc2** .  Ref: 9398669 Mol Biol Cell, 1997
- The pop1 mutation shows a synthetic lethal interaction with the temperature sensitive  **cdc2**  or  **cdc13**  mutation.  Ref: 9203581 Genes Dev, 1997
- Nuclear  **cdc2**  YFP fluorescence oscillates with that of  **cdc13**  YFP cyclin.  Ref: 11683390 J Cell Sci, 2001
- A specific synthetic peptide antibody generated to P. carinii  **Cdc13**  further demonstrated differential  **Cdc2**   **Cdc13**  activity over the life cycle of P. carinii, with greater activity in cysts compared with trophic forms of the organism.  Ref: 10837370 Am J Respir Cell Mol Biol, 2000
- At the G2 M boundary, the Cdc25 protein phosphatase is activated to dephosphorylate the  **Cdc2**   **Cdc13**  complex, resulting in abrupt activation of  **Cdc2**  kinase activity and entry into mitosis.  Ref: 9552387 Prog Cell Cycle Res, 1996
- The meta anaphase transition is controlled by interactions between  **Cdc13**   **Cdc2**  and the anaphase promoting complex, which labels  **Cdc13**  subunits for proteolysis.  Ref: 9652094 Biophys Chem, 1998
- Here, we characterize  **cdc2**  5w and two alleles of  **cdc13** , which have implications for the understanding of details of the interactions amongst  **Cdc2** ,  **Cdc13** , and Wee1.  Ref: 8757394 Mol Gen Genet, 1996
- One operates through the  **Cdc2**   **Cdc13**  kinase, which also involves the CDK  [ **CDC2** ]  inhibitor Rum1, and the other through the S phase regulator Cdc18.  Ref: 9203581 Genes Dev, 1997
- The M phase induction activity of  **Cdc2**   **Cdc13**  is inhibited by Wee1 tyrosine kinase, which phosphorylates  **Cdc2**  on tyrosine 15.  Ref: 8972180 Mol Cell Biol, 1997

**Non-interactive relationship** (e.g. studied together, co-existance, homology, etc.)

- In opposition to these cyclins the rum1 inhibitor, a protein present exclusively in G1, prevents premature activation of the  **cdc2**  cig2 and the  **cdc2**   **cdc13**  complexes until cells have reached the critical cell size required to pass Start and initiate a new cell cycle.  Ref: 9552380 Prog Cell Cycle Res, 1996
- **Cdc2** ,  **Cdc13** , and Cdc25 act as positive regulators of cell cycle progression at the G2 M boundary, while Wee1 and Miky1 play a negative regulatory role.  Ref: 8757394 Mol Gen Genet, 1996
- This process is dependent on Rum1, an inhibitor of the  **Cdc2**   **Cdc13**  and  **Cdc2**  Cig2 cyclin B kinases.  Ref: 8972180 Mol Cell Biol, 1997
- Cdc18 overexpression induces DNA rereplication without mitosis, as does elimination of  **Cdc2**   **Cdc13**  kinase during G2 phase.  Ref: 9436991 Mol Biol Cell, 1998
- The M phase inducing  **Cdc2**   **Cdc13**  cyclin dependent kinase is inhibited by Wee1 tyrosine kinase and activated by Cdc25 phosphatase.  Ref: 8930897 Mol Biol Cell, 1996
- Although we did not identify such molecules, we isolated a number of alleles of both  **cdc2**  and  **cdc13** , including a novel wee allele of  **cdc2** ,  **cdc2**  5w.  Ref: 8757394 Mol Gen Genet, 1996
- We propose that the intracellular distribution of Nim1 and Wee1 may serve to coordinate the regulation of nuclear  **Cdc2**   **Cdc13**  with cytoplasmic growth.  Ref: 8930897 Mol Biol Cell, 1996
- A detailed mathematical model for the fission yeast mitotic cycle is developed based on positive and negative feedback loops by which  **Cdc13**   **Cdc2**  kinase activates and inactivates itself.  Ref: 10884416 Proc Natl Acad Sci U S A, 2000
- An mfr1 null mutant completes meiosis II but remains with high levels of  **cdc13**  and  **cdc2**  kinase activity and has considerably delayed spore formation.  Ref: 11493649 J Cell Sci, 2001
- Consistent with this hypothesis, we report that Cdc18 interacts with  **Cdc2**  in association with  **Cdc13**  and Cig2 B type cyclins in vivo.  Ref: 9436991 Mol Biol Cell, 1998
- A novel protein, Psp1, essential for cell cycle progression of Schizosaccharomyces pombe is phosphorylated by  **Cdc2**   **Cdc13**  upon entry into G0 like stationary phase of cell growth.  Ref: 9242669 J Biol Chem, 1997
- **Cdc2**  YFP and  **cdc13**  YFP are highly enriched on the spindle pole body of cells in late G2 or arrested at S phase.  Ref: 11683390 J Cell Sci, 2001
- Surprisingly, Csk1 also activated  **Cdc2**  in complexes with either  **Cdc13**  or Cig2 cyclins.  Ref: 10226032 Curr Biol, 1999
- The  **Cdc2**   **Cdc13**  complex is shown to phosphorylate the GST Psp1 fusion protein in vitro, and site directed mutagenesis and phosphoamino acid analysis indicated that the serine residue at position 333 in the carboxyl terminal region is required for phosphorylation.  Ref: 9242669 J Biol Chem, 1997
- The onset of mitosis is governed by  **cdc2**  in partnership with the B type cyclin,  **cdc13** .  Ref: 8855663 Trends Genet, 1996
- In the model organism Schizosaccharomysces pombe, potentially active  **Cdc2**   **Cdc13**  kinase complex accumulates throughout the S and G2 phases of the cell cycle.  Ref: 9552387 Prog Cell Cycle Res, 1996
- A slow negative feedback loop is turned on during mitosis by activation of Slp1 anaphase promoting complex APC, which indirectly re activates the negative regulators, leading to a drop in  **Cdc13**   **Cdc2**  activity and exit from mitosis.  Ref: 10884416 Proc Natl Acad Sci U S A, 2000
- **Cdc2**   **Cdc13**  activity is also indirectly regulated by the approximately 70 kDa Nim1 Cdrl serine threonine kinase, which promotes mitosis by inhibiting Wee1 via direct phosphorylation.  Ref: 9135149 EMBO J, 1997
- When starved for nitrogen source, they fail to effectively arrest in G1 and die of accelerated mitotic catastrophe if regulation of p34  **cdc2**   **Cdc13**  by inhibitory tyrosine phosphorylation is compromised by partial inactivation of Wee1 kinase.  Ref: 9398669 Mol Biol Cell, 1997
- Interestingly, Cdc6 is an in vitro substrate for  **Cdc13**   **Cdc2**  and Cig1  **Cdc2** , but not for Cig2  **Cdc2**  associated kinases.  Ref: 10381393 J Cell Sci, 1999
- Previous studies showed that the  **Cdc2**   **Cdc13**  complex is located in the nucleus.  Ref: 8930897 Mol Biol Cell, 1996
- This mitotic induction operates, at least in part, through activation of the normal G 2 M CDK  [ **CDC2** ]  complex  **Cdc13**   **Cdc2** .  Ref: 12093738 EMBO J, 2002
- All events of the fission yeast cell cycle can be orchestrated by fluctuations of a single cyclin dependent protein kinase  [ **CDC2** ] , the  **Cdc13**   **Cdc2**  heterodimer.  Ref: 9652094 Biophys Chem, 1998
- We show that in fission yeast the mitotic B type cyclin  **Cdc13**   **Cdc2**  kinase associates with replication origins in vivo.  Ref: 12419251 Cell, 2002
- To further study the regulation of the life cycle of P. carinii, we characterized the P. carinii B type cyclin termed  **Cdc13** , whose binding to  **Cdc2**  is necessary for kinase activity.  Ref: 10837370 Am J Respir Cell Mol Biol, 2000
- Novel alleles of  **cdc13**  and  **cdc2**  isolated as suppressors of mitotic catastrophe in Schizosaccharomyces pombe.  Ref: 8757394 Mol Gen Genet, 1996
- Recent evidence shows that the  **cdc2**   **cdc13**  complex can also control the onset of S phase and, in addition, ensures that there is only one S phase per cell cycle.  Ref: 8855663 Trends Genet, 1996
- To investigate the mechanism coupling growth protein synthesis with cell division, we examined the relationship between the tyrosine kinase Wee1 that inhibits  **Cdc2**   **Cdc13**  mitosis inducing kinase by phosphorylating it, and protein synthesis inhibition in fission yeast.  Ref: 11119724 FEBS Lett, 2000
- Cell cycle control in the fission yeast Schizosaccharomyces pombe involves interplay amongst a number of regulatory molecules, including the  **cdc2** ,  **cdc13** , cdc25, wee1, and mik1 gene products.  Ref: 8757394 Mol Gen Genet, 1996
- Positive feedbacks are created by  **Cdc13**   **Cdc2**  dependent phosphorylation of specific substrates inactivating its negative regulators Rum1, Ste9 and Wee1 Mik1 and activating its positive regulator Cdc25.  Ref: 10884416 Proc Natl Acad Sci U S A, 2000
- These findings identify Cdc18 as a key target of  **Cdc2**   **Cdc13**  and  **Cdc2**  Cig2 kinases in the mechanism that limits chromosomal DNA replication to once per cell cycle.  Ref: 9436991 Mol Biol Cell, 1998
- The model explains how fission yeast cells can exit mitosis in the absence of Ste9  **Cdc13**  degradation and Rum1 an inhibitor of  **Cdc13**   **Cdc2** .  Ref: 10884416 Proc Natl Acad Sci U S A, 2000
- In Schizosaccharomyces pombe, the activity of the M phase inducing  **Cdc2**   **Cdc13**  cyclin dependent kinase is inhibited by Wee1 and Mik1 tyrosine kinases, and activated by Cdc25 and Pyp3 tyrosine phosphatases.  Ref: 9135149 EMBO J, 1997
- If cdc13p cannot be recognized by the anaphase promoting complex,  **cdc2**  YFP and  **cdc13**  YFP remain associated with the spindle.  Ref: 11683390 J Cell Sci, 2001
- In fission yeast, the length of the G1 phase depends on a precise balance between levels of positive cig1, cig2, puc1, and  **cdc13**  cyclins and negative rum1 and ste9 APC regulators of  **cdc2** .  Ref: 10679013 Mol Biol Cell, 2000
